# Stimulating Self-Regulation: A Review of Non-invasive Brain Stimulation Studies of Goal-Directed Behavior

**DOI:** 10.3389/fnbeh.2018.00337

**Published:** 2019-01-18

**Authors:** Nicholas J. Kelley, Alessia Gallucci, Paolo Riva, Leonor Josefina Romero Lauro, Brandon J. Schmeichel

**Affiliations:** ^1^Department of Psychology, Northwestern University, Evanston, IL, United States; ^2^Department of Psychology, University of Milano-Bicocca, Milan, Italy; ^3^Department of Psychological and Brain Sciences, Texas A&M University, College Station, TX, United States

**Keywords:** transcranial direct current stimulation, self-regulation, emotion-regulation, goal-directed behavior, dorsolateral prefrontal cortex

## Abstract

Self-regulation enables individuals to guide their thoughts, feelings, and behaviors in a purposeful manner. Self-regulation is thus crucial for goal-directed behavior and contributes to many consequential outcomes in life including physical health, psychological well-being, ethical decision making, and strong interpersonal relationships. Neuroscientific research has revealed that the prefrontal cortex plays a central role in self-regulation, specifically by exerting top-down control over subcortical regions involved in reward (e.g., striatum) and emotion (e.g., amygdala). To orient readers, we first offer a methodological overview of tDCS and then review experiments using non-invasive brain stimulation techniques (especially transcranial direct current stimulation) to target prefrontal brain regions implicated in self-regulation. We focus on brain stimulation studies of self-regulatory behavior across three broad domains of response: persistence, delay behavior, and impulse control. We suggest that stimulating the prefrontal cortex promotes successful self-regulation by altering the balance in activity between the prefrontal cortex and subcortical regions involved in emotion and reward processing.

## Introduction

At least since Walter Mischel’s seminal work on delay of gratification (Mischel, 1958; Mischel et al., 1972), the practical and the theoretical implications of self-regulation have been important topics of study in psychological science (Carver and Scheier, 1982; Metcalfe and Mischel, 1999; Muraven and Baumeister, 2000). Self-regulation refers to the conscious and non-conscious processes that enable individuals to guide their thoughts, feelings, and behaviors in a purposeful manner. Self-regulation is crucial for goal-directed behavior and has been related to many consequential outcomes in life including physical and mental health, psychological well-being, ethical decision making, and strong interpersonal relationships. Likewise, failures at self-regulation are thought to contribute to alcohol and drug addiction, personal debt, obesity, and other outcomes that carry both personal and societal costs (for a review, see Vohs and Baumeister, 2016). Because of its consequences for so many crucial outcomes, the scientific study of self-regulation spans many of subfields in psychological science and frequently centers on a relatively simple question: How can we improve self-regulatory abilities?

One possible answer is by stimulating the brain with electrical current. Self-regulation is typically assessed with laboratory analogs of common challenges encountered in daily life, such as persisting at difficult tasks, choosing between immediate versus more delayed rewards, and managing emotional impulses. These examples represent paradigmatic forms of self-regulation, namely *persistence*, or the sustained performance of aversive behavior (e.g., tolerating pain, coping with failure); *delay behavior*, which refers to choices that favor more long-term investments at the expense of short-term gains; and *impulse control*, which involves the purposeful inhibition of emotive response tendencies. Below we focus on these three paradigmatic forms of self-regulation and review experiments that have used brain stimulation techniques to try to improve them. We then consider inconsistencies in findings, address unresolved questions, and point to new directions for future research.

### Neural Correlates of Self-Regulation

Convergent evidence from social, cognitive, and affective neuroscience research reveals that the interplay between the prefrontal cortex and subcortical threat and reward processing is crucial for self-regulation (for a review see: Heatherton and Wagner, 2011; Kelley et al., 2015b; Berkman, 2017; Wagner and Heatherton, 2017). Numerous studies have associated successful self-regulation with top-down control from the prefrontal cortex over subcortical regions involved in reward and threat processing (e.g., Dambacher et al., 2014; Giuliani et al., 2014; Vijayakumar et al., 2014; Lopez et al., 2017). By contrast, self-regulatory failure occurs when top-down control is diminished or when the balance in activity favors threat and reward systems (e.g., Demos et al., 2012; Wagner and Heatherton, 2012; Wagner et al., 2013; Chester and DeWall, 2014; Lopez et al., 2014; Meyer and Bucci, 2016).

As an illustrative example, in a 40-year longitudinal follow-up with children who participated in Mischel’s seminal delay of gratification work, children who successfully delayed gratification exhibited preferential recruitment of the prefrontal cortex (PFC) during a task requiring inhibitory control as adults. In contrast, children who were unsuccessful at delaying gratification showed preferential recruitment of the ventral striatum in a delay task as adults (Casey et al., 2011). These findings highlight the importance of the balance or relative activity levels in the prefrontal cortex and subcortical regions, respectively, and how shifts in this balance can tip the scales between short-term and long-term goal regulation.

Several models of emotion regulation similarly emphasize the balance in activity between prefrontal and subcortical brain regions. For example, Ochsner and Gross (2007) proposed a seminal model of emotion that distinguishes between bottom-up affective processing, mediated by limbic system structures such as amygdala, and top-down appraisal processes that involve prefrontal regions. Success at cognitive reappraisal, a form of emotion regulation that involves generating cool cognitive representations of affective states or changing the meaning of emotional events, is associated with increased activity in dorsomedial and dorsolateral prefrontal cortices, alongside corresponding decreases limbic system (i.e., amygdala) activity.

Another relevant model of emotion regulation highlighted the key role of a cortico-subcortical network consisting in a dorsal system and a ventral system (Phillips, 2003; Phillips et al., 2008). The dorsal system encompasses the hippocampus, the dorsal regions of anterior cingulate gyrus, and the DLPFC and is involved in executive functions, particularly planning, attention control, and effortful regulation of affective states. The ventral system includes the amygdala, the insula, the ventral striatum, the anterior cingulated cortex, the orbitofrontal cortex, and the ventrolateral prefrontal cortex (VLPFC) and is predominantly recruited for the automatic regulation of affective reactions and for the recognition of emotional valence of stimuli.

Consistent with and prior to these models of emotion regulation, Botvinick and colleagues (Cohen et al., 2000; Botvinick et al., 2001, 2004; Shenhav et al., 2013) discussed cortical-subcortical balance as it relates to cognitive control. Cognitive control refers to a constellation of functions that orient cognitive subsystems to perform difficult and novel tasks (Botvinick et al., 2004). Botvinick and colleagues’ conflict monitoring hypothesis proposed that response conflict activates cortical brain regions (i.e., the dorsal ACC), thereby signaling the need for conflict resolution to facilitate effective behavior (see MacDonald et al., 2000; Botvinick et al., 2001; Ochsner et al., 2009). The theoretical models reviewed above suggest that regulation of both cognition and emotion is contingent upon the balance in activity between cortical and subcortical regions. Brain stimulation techniques targeting cortical regions may thus influence self-regulation by influencing cortical-subcortical balance.

### Overview and Goals

We propose that non-invasive brain stimulation targeting the prefrontal cortex holds promise for improving human self-regulation. To orient the reader to neuromodulation, we first provide a methodological overview of transcranial direct current stimulation (tDCS) – one of the most popular brain stimulation techniques in psychological science. Then we review behavioral evidence that non-invasive brain stimulation targeting the PFC may enhance three paradigmatic forms of self-regulation: persistence, delay behavior, and impulse control (see Table 1). We conclude our review by highlighting inconsistencies in findings, unresolved questions, and new directions for future research.

**Table 1:** Study features, stimulation parameters, and key outcomes of all studies reviewed.

N	Time (min)	Size (cm^2^)	Current (mA)	Montage	Design	Outcome
**Persistence**				
20	5	35	2	Sham, anode L DLPFC, M1, V1	W	Anode L DLPFC increased pain thresholds (Boggio et al., 2008)
12	20	35	2	Sham, anode and cathode tDCS over L and R DLPFC	W	Anode R DLPFC increased tolerance to heat (Mylius et al., 2012)
40	20	15	2	Anode and cathode tDCS over the L and R DLPFC	W	Anode L DLPFC decreased pain (Mariano et al., 2015)
79	20	16	2	Sham, anode L DLPFC, and cathode L DLPFC	B	Cathode L DFLPFC increased pain tolerance (Powers et al., 2018)
**Delay behavior**				
14	20	9	1.6	Sham, anode L DLPFC/cathode R DLPFC, cathode L DLPFC/anode R DLPFC	W	Anode L DLFPC increased preference for immediate rewards (Hecht et al., 2012)
24	20	35	1.5	Sham, anode L DLPFC/cathode R OFC, cathode L DLPFC/anode R OFC	W	Both types of active stimulation increased preference for larger-but-later rewards (Nejati et al., 2018)
23	20	35	2	Sham, anode L DLPFC/cathode R DLPFC, and cathode L DLPFC/anode R DLPFC	W	Anode R DLPFC decreased food cravings, visual attention toward desserts, and consumption (Fregni et al., 2008)
19	20	35	2	Sham and cathode L DLPFC/anode R DLPFC	W	Anode R DLPFC decreased food cravings but not consumption (Goldman et al., 2011)
10	20	35	2	Sham and cathode L DLPFC/anode R DLPFC	W	Anode R DLPFC reduced consumption and modulated the N2, P3a, and P3b ERP components (Lapenta et al., 2014)
17	20	25	2	Sham and cathode L DLPFC/anode R DLPFC	W	Anode R DLPFC reduced cravings for sweet but not savory foods or consumption (Kekic et al., 2014)
30	20	35	2	Daily sham or anode R DLPFC for 5 days	B	Anode R DLPFC reduced food cravings up to 30 days later (Ljubisavljevic et al., 2016)
30	20	25	2	Sham and cathode L DLPFC/anode R DLPFC	W	Anode R DLPFC reduced cravings and consumption (Burgess et al., 2016)
**Impulse control**				
20	15	5.3	0.45	Sham, anode L DLPFC, and anode R DLPFC	W	Anode R DLPFC combined with a cognitive reappraisal task reduced negative emotions (Pripfl and Lamm, 2015)
96	20	25/35	1.5	Sham or anode R VLPFC	B	Anode R VLPFC reduced the perceived intensity of negative emotions (Vergallito et al., 2018)
60	15	35	2	Sham, cathode L DLPFC/anode R DLPFC, or anode L DLPFC/cathode R DLPFC	B	Anode L DLPFC caused more aggression in angry participants (Hortensius et al., 2012)
32	12.5	35	2	Sham or anode R DLPFC	B	Anode R DLPFC reduced proactive aggression in men (Dambacher et al., 2014)
64	21.75	35	1.5	Sham, cathode L IFG/anode R IFG, and anode L IFG/cathode R IFG	B	No effect of IFG stimulation on response inhibition or aggression (Dambacher et al., 2015b)
90	15	35	2	Sham, cathode L DLPFC/anode R DLPFC, and anode L DLPFC/cathode R DLPFC	B	Anode R DLPFC increased rumination (Kelley et al., 2013)
202	15	35	2	Sham, cathode L DLPFC/anode R DLPFC, and anode L DLPFC/cathode R DLPFC	B	Anode R DLPFC sped up motive incongruent responses (Kelley and Schmeichel, 2016)
14	20	32	2	Five days of twice daily anode L DLPFC/cathode R DLPFC	N/A	Anode L DLPFC reduced sadness up to 30 days after treatment (Ferrucci et al., 2009)
10	20	35	1	Daily sham or anode L tDCS	B	Anode L DLPFC reduced depressive symptoms (Fregni et al., 2006)
23	5	35	2	Sham, anode L DLPFC, M1, V1	W	Anode L DLPFC increased accuracy in identifying positive pictures (Boggio et al., 2009)
80	20	35	2	Sham and anode R IFG	B	Anode R IFG decreased sustained fear and skin conductance levels to unpredictable threats (Herrmann et al., 2017)
45	30	25	1	Sham and either cathode L DLPFC/anode R DLPFC, or anode L DLPFC/cathode R DLPFC	W	Anode L DLPFC improved performance and decreased cortisol among participants high in ma anxiety (Sarkar et al., 2014)
47	20	25	1	Sham and anode L DLPFC	B	Anode L DLPFC enhanced fear memories (Mungee et al., 2014)
17	20	25	1	Sham and cathode R DLPFC	B	No effect of cathode R DLPFC on fear memories (Mungee et al., 2016)
80	20	25/35	1.5	Sham and anode R VLPFC	B	Anode R VLPFC reduced aggression after social exclusion (Riva et al., 2012)
79	15	25/35	1.5	Sham and anode R VLPFC	B	Anode R VLPFC reduced negative feelings after social exclusion (Riva et al., 2014a)
	20	25/50	1.5	Sham and anode R VLPFC	B	Anode R VLPFC reduced unproved aggression in violent-game players (Riva et al., 2017)
92	15	35	2	Sham, cathode L DLPFC/anode DLPFC, and anode L DLPFC/cathode R DLPFC	B	Anode L DLPFC increased jealousy after social exclusion (Kelley et al., 2015a)
16	20	35	1	Sham and anode L DLPFC	W	Negative pictures rated less negative after anode tDCS over the L DLPFC (Peña-Gómez et al., 2011)
48	20	35/100	1.5	Sham and anode R DLPFC	B	Anode R DLPFC enhanced cognitive control during emotion regulation (Feeser et al., 2014)
35/12	20	35	2	S1: Bilateral DLPFC S2: Unilateral DLPFC	B	Bilateral DLPFC tDCS modulated decision making (Fecteau et al., 2007b)
36		35	2	Sham, cathode L DLPFC/anode DLPFC, and anode L DLPFC/cathode R DLPFC	B	Anode R DLPFC reduced risk taking (Fecteau et al., 2007a)
16	19	35	2	Sham, cathode DLPFC/anode R DLPFC, and anode L DLPFC/cathode R DLPFC	W	Anode stimulation over the R DLPFC reduced risky decision-king (Cheng and Lee, 2016)
30	25	25	2	Twice daily sham or anode R DLPFC/cathode L DLPFC for 5 days	B	Active tDCS paired with the cognitive task reduced risk-taking This effects persisted 2 months (Gilmore et al., 2017)
20	15	25	1.5	Sham or anode R DLPFC/cathode L DLPFC	B	Anode R DLPFC caused greater R DLPFC-whole brain connectivity which was associated with reduced risk-taking (Wacker et al., 2008)
24	15	35	1	Anode and cathode R DLPFC or anode and cathode L DLPFC	W	Anode DLPFC (either L or R) led to reduced risk taking on a driving simulation (Beeli et al., 2008)

*DLPFC, dorsolateral prefrontal cortex; VLPFC, ventrolateral prefrontal cortex; L, left; R, right; B, between-subjects; W, within-subjects.*

## A Methodological Overview of tDCS

Effects of weak electrical currents on brain and neuronal function were first described more than two centuries ago (Priori, 2003; Nitsche et al., 2008; Zago et al., 2008). Systematic studies with animals showed the efficacy of inducing modifications, enhancements, or diminutions of cortical activity by delivering weak direct currents to the brains of laboratory rats. Specifically, passing currents through the scalp polarized the brain region beneath the electrodes and altered the firing rate of neurons. These effects were detected immediately after the stimulation and seemed to last beyond the stimulation period (Bindman et al., 1964; Gartside, 1968; Hattori et al., 1990; Islam et al., 1995). Early research in human samples investigated the application of electrical currents in mood disorders treatment, with some evidence suggesting a reduction in symptoms of depression and mania (Costain et al., 1964; Carney, 1969). But subsequent studies and null findings contributed to skepticism in the efficacy of running weak electrical currents into the brain as an effective tool for symptom reduction (Lolas, 1977; Nitsche et al., 2003b). More recently, researchers have shed light on bidirectional, time, and polarity-dependent excitability changes following tDCS (Priori et al., 1998; Nitsche and Paulus, 2000).

### How Does tDCS Work?

The tDCS device consists of an electric stimulator, which delivers a constant current and an isolation current, linked to a pair of electrodes positioned on the scalp over cortical regions of interest. The electrodes, namely an anode and a cathode, are typically covered by sponges soaked in NaCI solution (or electrode cream) to increase conductivity, reduce resistance, and improve the homogeneity of the electric field under the electrodes.

Transcranial direct current stimulation differs from other brain stimulation techniques, such as transcranial electrical stimulation (TES) and transcranial magnetic stimulation (TMS), because tDCS does not induce action potentials in neuronal membrane. Instead, tDCS transiently modifies spontaneous neuronal excitability by depolarizing or hyperpolarizing neurons’ resting membrane potentials, producing ionic concentration shifts within the extracellular fluid (Creutzfeldt et al., 1962; Purpura and McMurtry, 1965). Anodal stimulation typically depolarizes local neurons, which in turn will require less dendritic input to fire, whilst cathodal stimulation hyperpolarizes neurons’ typical resting membrane potentials so that increasing dendritic input is required (Nitsche and Paulus, 2000). This mechanism of action generally occurs both during stimulation and for a short period of time (<5 min) thereafter.

Transcranial direct current stimulation has been found to involve more complex mechanisms including long-term potentiation (LTP) and long-term depression (LTD) mechanisms at the synaptic level, affecting hyper-communicative activity through the anode and hypo-communicative activity through the cathode. These tDCS-driven changes in LTP and LTD may be mediated by a number of synaptic mechanisms including: NMDA (*N*-methyl-D-aspartate) receptors, GABAergic activity, glutamatergic activity, intracellular CA2+ concentration, brain-derived neurotrophic factor (BDNF) secretion, and tropomyosin-related kinase B (TrkB) activation (Liebetanz et al., 2002; Nitsche et al., 2003a, 2004; Stagg et al., 2009; Fritsch et al., 2010; Stagg and Nitsche, 2011). Also, non-synaptic mechanisms, such as changes in pH and transmembrane proteins, seem to be involved in long-term effects of tDCS (Ardolino et al., 2005).

### Parameters Influencing Stimulation Efficacy

The efficacy of eliciting changes in brain activity using tDCS depends on several physical parameters including current density, stimulation duration, and the orientation and focality of the electrical field. These parameters constitute the tDCS dosage.

#### Current Density

Current density represents the ratio between current strength (normally up to 2 mA) and electrode size (normally reference electrode varies from 25 to 35 cm^2^). Current density determines the delivered electrical field strength (Purpura and McMurtry, 1965).

#### Stimulation Duration

Stimulation duration refers to the amount of time participants undergo stimulation. It is based on LTP and LTD mechanisms and is related to the occurrence and length of aftereffects. Generally, keeping current density constant, brief exposure to tDCS stimulation (few seconds) does not induce long-lasting effects, whereas tDCS stimulations of about 10 min (up to 30 min) typically do elicit aftereffects (Nitsche et al., 2003c; Ardolino et al., 2005).

#### Orientation of the Electric Field

The orientation of the electric field normally depends on electrodes’ polarity and position. As already described, tDCS produces polarity-dependent effects whereby anodal stimulation increases the activity of the stimulated area, whereas cathodal stimulation decreases it. Several studies of both the primary motor and visual cortices have found that different electrode positions modulate different neuronal groups and elicit different evoked potentials (Priori et al., 1998; Antal et al., 2004; Accornero et al., 2007) suggesting that electrode position is a crucial tDCS parameter.

Indeed, not only may electrode position affect the amount of current delivered to the brain and the direction of current flow, but also it may determine effects on the targeted brain region, due to electrical field interactions associated with neuronal geometry (Nitsche and Paulus, 2000; Nitsche et al., 2008). Whereas the coupling of anodal-excitatory and cathodal-inhibitory effects is well established in the sensorimotor domain, evidence gets more controversial when addressing higher cognitive functions (Jacobson et al., 2012). This lack of consistency between sensorimotor and cognitive functions is at this output level. It is not that the inconsistencies arise from differences in the effects of stimulation on sensorimotor cortices versus prefrontal cortices themselves. In other words, the inconsistency is not the result in differences in the effects of the stimulation protocol on activity or function but rather the consequence of those changes. Indeed, when dealing with more complex functions, likely represented by large and interconnected neural networks comprising both excitatory and inhibitory connections, it is more difficult to obtain a predictable outcome, hence it is not always the case that anodal tDCS leads to an enhancement (e.g., better performance in a task) and cathodal tDCS leads to a diminution of the assessed cognitive function (Fertonani and Miniussi, 2017).

#### Induced Electric Field Focality

Another important tDCS parameter is the induced electric field focality. Generally, large electrodes and bipolar scalp electrode arrangements limit tDCS focality (Gandiga et al., 2006), in part because large electrodes may alter activity in areas adjacent to the stimulated region. Moreover, with an intracephalic montage, the so-called reference electrode (the secondary electrode with regard to a specific experimental setting), being located on the scalp, is not entirely inert. The lack of spatial focality of tDCS effects suggests the need for arrangements to increase focality. For instance, to increase tDCS focality it is possible to use smaller target electrodes. Another option is to increase the reference electrode size so that, due to decreased current density, it becomes practically inert. Alternatively, using an extracephalic montage may increase stimulation focality (Nitsche et al., 2007, 2008; Ferrucci et al., 2008), although in this case the spread of current flow could be hardly traceable. In any case, widespread (non-focal) effects of tDCS should be taken into account, considering that functionally active cortical targets may be more susceptible to excitability changes induced by tDCS. Lack of spatial focality may undermine tDCS effectiveness in performing cortical mapping, but the idea that widespread cortical networks are affected by tDCS could explain the strength of the observed behavioral effects, in some cases lasting for months after several stimulation sessions are performed, thus supporting the possible benefits of this technique for clinical applications.

#### Underlying Neural Activity

Studies using fMRI to assess online and offline tDCS effects have found that anodal and cathodal stimulation elicit, respectively, an increment and a decrement of perfusion in a wide set of brain areas, including cortical and subcortical structures, even at some distance from the target area (e.g., Stagg et al., 2013). Similarly, computational models of current flow have indicated that strong electric fields occur not only underneath and near the stimulating electrodes but also in the regions between them (Miranda et al., 2013). Consistent with the computational modeling studies noted above, recent findings have observed tDCS effects on both structural and functional connectivity (Romero Lauro et al., 2014, 2016; Pisoni et al., 2017).

### Practical Considerations

#### Sham Stimulation

Regarding its practical applications, tDCS, allows for more effective placebo stimulation-controlled studies compared to TMS where notable issues with placebo effects and limited blinding success (Duecker and Sack, 2015). Placebo (sham) stimulation can be delivered for few seconds and subjects experience the same physical sensations as real stimulation (e.g., itching sensation), without substantial neural or behavioral effects (Gandiga et al., 2006; Nitsche et al., 2008).

#### Individual Differences

Another practical consideration concerns individual differences. Following seminal work in the tDCS literature (e.g., Nitsche et al., 2008) we recommend that participants be matched on individual differences like sex or age that can influence tDCS efficacy (Pitcher et al., 2003; Kuo et al., 2006; Quartarone et al., 2007; Chaieb et al., 2008). In addition to demographic factors, researchers should also consider whether participants respond to tDCS protocols. In two motor cortex studies, anodal stimulation increased cortical excitability in 50–64% of participants (Wiethoff et al., 2014; López-Alonso et al., 2015). These individual differences highlight the need to develop more robust stimulation protocols and well-powered studies which can systematically test for the moderating role of individual differences.

#### Safety

Transcranial direct current stimulation is a safe and non-invasive neuromodulatory technique. Several neuroimaging and EEG studies have demonstrated that tDCS does not cause adverse effects on the brain (Nitsche et al., 2004; Iyer et al., 2005). The most common side effects include mild tingling sensations, moderate fatigue and light itching sensations, especially at stimulation onset (e.g., Poreisz et al., 2007) and stimulation sessions up to 50 min do not cause serious consequences (Nitsche et al., 2008). Moreover, ramping up and ramping down current at the beginning and at the end of a stimulation session is useful to avoid brief retinal phosphenes or startle-like phenomenon caused by sudden neuronal firings.

To summarize, tDCS is a safe, inexpensive, well-tolerated, and easy to use. Its physiological consequences have led to an increasing interest in using tDCS to alter psychological processes. With an appreciation for the history and mechanics of tDCS, the next section will explore the consequences of prefrontal tDCS psychological processes in the domain of self-regulation.

## Transcranial Direction Current Stimulation and Self-Regulation

### Persistence

Self-regulation enables individuals to guide their thoughts, feelings, and behaviors in a goal-directed fashion. Self-regulation can be aversive both physically and psychologically. Goal directed behavior often entails persistence, including the sustained performance of aversive behavior. For example, physical exercise sometimes elicits short-term pain or physical discomfort but also brings more long-term appearance- and health-related benefits. Individuals who are better able to endure the discomfort presumably exercise longer and more frequently. As a result, these same individuals may be more successful at achieving their fitness goals. Other situations may require individuals to endure psychologically aversive states in the interest of accomplishing one’s goals. For example, academic and occupational successes may entail many failed attempts to solve complex problems. The ability to endure these aversive states seems likely to facilitate goal-directed behavior and thus may constitute an important facet of self-regulation.

#### Pain Tolerance

Research suggests that the DLPFC is a key brain region for various aspect of pain, including pain tolerance (see Seminowicz and Moayedi, 2017). Consistent with this viewpoint, several studies have used prefrontal tDCS to modulate the experience of pain in laboratory tasks. For example, Mylius et al. (2012) found that anodal tDCS over the right DLPFC increases tolerance to heat pain as measured by the temperature of a thermode applied to the forearm. Similar results were obtained by Boggio et al. (2008), who found that excitatory stimulation of the left DLPFC increases pain thresholds. Taken together, these findings suggest that stimulation to the prefrontal cortex in either hemisphere may increase pain tolerance – a classic form of self-regulation.

More recently, a study by Mariano et al. (2016) applied anodal versus sham tDCS during both a cold pressor task and a breath holding task (see also Mariano et al., 2015, for similar work probing the dorsal anterior cingulate cortex). These two tasks represent commonly used laboratory pain paradigms. Participants’ pain ratings were assessed before and after stimulation using the Defense and Veterans Pain Rating Scale (DVPRS), which asks participants to rate their pain on an 11-point visual analog scale from 0 = *no pain* to 10 = *severe pain*. Ratings were obtained after the first 7 min of tDCS in each testing block and immediately after the cold pressor and breath holding tasks. Stimulation did not influence performance on the cold pressor task as measured by threshold, tolerance, or endurance, nor did it influence breath holding time. However, anodal stimulation over the left DLPFC decreased the experience of pain as measured by the DVPRS. This study suggests that excitatory stimulation of the DLPFC may influence pain perception.

Another relevant pilot study by Powers et al. (2018) paired anodal, cathodal, or sham stimulation over the left DLPFC with either pain education or a 3-min audio recording designed to mimic key components of Cognitive-Behavioral Therapy for pain. Afterward, participants completed five trials of a heat tolerance pain test. Regardless of which intervention was paired with tDCS, cathodal stimulation over the left DFLPFC increased pain tolerance. But several limitations in this study mar the interpretability of the results. First, the study included 6 experimental conditions with 79 total participants, resulting in small sample sizes in each condition and thus relatively low statistical power to detect anything but very large effects of tDCS. Second, the electrode montage involved placing the anode or cathode over the F3 region and the other electrode on the right shoulder. The study by Mariano et al. (2016) reviewed above, which found reduced pain experience but not increased pain tolerance, used the mastoid as a reference, whereas the study by Mylius and colleagues, which found increased pain tolerance, involved placing the reference electrode over the contralateral supraorbital area. Thus, inconsistencies in the electrode montages used across studies of prefrontal tDCS and pain, along with differences in methods used to induce and measure pain, hamper our ability to draw clear conclusions about the effects of tDCS on pain experience and pain tolerance.

### Delay Behavior

Often one’s end goals are situated far in the future, and pursuing such goals comes at the cost of satisfying more immediate desires. Saving for the future comes at the cost of spending today. Maintaining a healthy physique comes at the expense of delicious desserts. Academic achievement often comes at the expense of fraternization. *Delay behavior* refers to choices that favor more long-term investments at the expense of short-term gains. To the extent that individuals stifle or subdue their immediate urges, the better they are in striving toward their long-term goals.

#### Delay Discounting

Delay discounting refers to the reduction in the present value of a reward with delayed receipt. The basic idea is that the valuation of rewards degrades over time. So, for example, gaining $10 today would typically be valued more than gaining $10 in a month from today. For each unit of time increase in the delay to receipt, the value of a reward decreases (or is discounted) by a non-fixed proportion. In other words, the effect of delay on value is not the same across the range of delays. At short delays value decreases less steeply, whereas at longer delays value degrades more steeply. A hyperbolic discounting function captures the pattern that at shorter delays reward valuation degrades less than it does at longer delay periods. The steepness of the slope within this hyperbolic model reflects the extent to which people prefer smaller-but-immediate (compared to larger-but-delayed) rewards. A steep slope (i.e., a larger hyperbolic *k*) reflects a stronger preference for smaller-but-immediate rewards. A less steep slope (i.e., a smaller hyperbolic *k*) reflects a stronger preference for larger-but-delayed rewards. Typically, a preference for smaller-but-immediate rewards is thought to reflect impulsivity (poor self-regulation) whereas a preference for larger-but-delayed rewards is thought to reflect self-control (good self-regulation).

Hecht et al. (2012) paired prefrontal tDCS with a delay discounting task. Specifically, participants received anodal right DLPFC/cathodal left DLPFC, cathodal right DLPFC/anodal left DLFPC, or sham stimulation. They observed a greater preference for smaller-but-sooner rewards when participants had received anodal stimulation over the left DLFPC, suggesting that this pattern of stimulation increased impulsivity or reduced self-regulation. Similar results have been obtained in TMS studies disrupting right DLPFC activity (Figner et al., 2010; Smittenaar et al., 2013). More recently, a study by Nejati et al. (2018) paired DLPFC stimulation with OFC stimulation prior to a delay discounting task. Specifically, participants received sham stimulation, anodal left DLPFC/cathodal right OFC, and cathodal left DLPFC/anodal right OFC. They observed that relative to sham stimulation, both active stimulation conditions caused a greater preference for delayed rewards as reflected in a smaller *k* value. The results of this study hinge upon the interaction between the DLPFC and OFC and as a result we cannot determine to what extent they were driven by the DLPFC (or OFC). Because of this, the study by Nejati and colleagues differs in a critical way from the discounting studies reviewed above as those studies speak moreso to the role of hemispheric asymmetry in discounting behavior. Thus, the majority of evidence here suggests that stimulation to shift the balance in neural activity toward the left prefrontal cortex increases delay discounting in a manner that suggests poorer self-regulation.

#### Food Choice

Food choice and eating entail delay behavior insofar as choosing nutritious foods (e.g., vegetables) over tempting ones (e.g., chocolate cake) represents a choice favoring long-term investments in health at the expense of a short-term hedonic gain. Fregni et al. (2008) compared excitatory right DLPFC stimulation (cathode over F3/anode over F4), excitatory left DLPFC stimulation (anode over F3/cathode over F4), and sham stimulation in the context of food. Self-report measures of food craving and craving in response to food in the laboratory were assessed before and after tDCS. Additionally, after tDCS, participants had their gaze patterns recorded while they viewed an array of nature scenes and images of tempting foods (e.g., desserts). Last, participants had the opportunity to ingest foods and the number of calories ingested was recorded. Results indicated that excitatory right DLPFC stimulation decreased food cravings, decreased visual attention toward tempting desserts, and decreased caloric consumption relative sham stimulation.

Goldman et al. (2011) conducted a similar experiment in a group of healthy individuals with frequent food cravings. Participants viewed food images from the International Affective Picture System (IAPS; Lang et al., 2008) before and after tDCS. Additionally, after stimulation, participants were free to eat a variety of tempting foods including chips, cookies, chocolate, and donuts. Consistent with the results of Fregni et al. (2008), Goldman and colleagues found that excitatory right DLPFC stimulation (cathode over F3/anode over F4) decreases food cravings, especially for sweets. Unlike the study by Fregni et al., however, the study by Goldman et al. did not find that tDCS influences food consumption. Thus, stimulation over the right prefrontal cortex appears to influence self-regulation in the context of desire for tempting foods but has seemingly inconsistent effects on consumption.

More recently, a study by Lapenta et al. (2014) found that excitatory stimulation over the right DLPFC reduces caloric ingestion and food intake (e.g., cakes and sweets). Additionally, after stimulation participants completed a GO/NO-GO task while EEG was recorded. Excitatory stimulation over the right DLPFC modulated the N2, P3a, and P3b ERP components. All three of these components have been implicated in successful inhibitory control during GO/NO-GO tasks (e.g., Albert et al., 2013). This study thus provides more direct evidence that tDCS over prefrontal cortex influences regulatory mechanisms, which in turn influence food choice behavior.

Kekic et al. (2014) compared excitatory right DLPFC stimulation (cathode over F3/anode over F4) to sham stimulation as participants completed both a food craving questionnaire and a food challenge task before and after stimulation. The food challenge task consisted of two parts. First, participants watched two short videos of tempting foods (e.g., chocolate). Next, each of the foods from the videos was made available to participants in the laboratory. At this point participants rated their desire to eat and emotional reactions to each of the presented foods. After stimulation, participants were left alone with the foods and instructed to eat what they would like while the experimenter was out of the room. Consistent with past research (e.g., Fregni et al., 2008; Goldman et al., 2011), excitatory right DLPFC stimulation reduced cravings for sweet but not savory foods on the food challenge task. Much like the findings from Goldman et al. these effects did not extend to influence actual consumption during the free eating portion of the task. Kekic et al. further reported that the tDCS-driven reductions in cravings were more pronounced among participants with low (versus high) delay discounting tendencies. This pattern suggests that it was easier to modulate food-related cravings in individual relatively low in impulsivity (high in self-control).

Ljubisavljevic et al. (2016) explored the consequences of repeated stimulation over the DLPFC on food craving. In their study, participants received excitatory right DLPFC stimulation for five consecutive days. Replicating past research, a single session of excitatory right DLPFC stimulation reduced the intensity of food craving. The effects of five consecutive days of stimulation reduced cravings both immediately and 30 days later. The craving reductions were most pronounced for fast foods and desserts. These effects were not moderated by participants’ weight at the beginning of the study, nor did the stimulation protocol influence weight assessed 30 days later. Another study using a repeated stimulation design found that stimulation for 8 straight days reduced both self-reported appetite and caloric consumption during a free eating buffet on the last day of stimulation (Jauch-Chara et al., 2014).

Inspired by the evidence linking excitatory right DLPFC stimulation to reductions in food cravings, Burgess et al. (2016) extended this body of research by testing a sample of participants with clinical or subclinical binge eating disorder. They observed that excitatory right DLPFC stimulation not only reduced cravings across food categories but also decreased desire to binge eat and decreased food consumption. In summary, research using tDCS to influence delay behavior has occasionally tested delay discounting for monetary rewards, and the evidence suggests that a left lateralized pattern of prefrontal stimulation induces a more impulsive preference for immediate rewards. Even more studies have concentrated on the domain of food craving and food consumption. Much of this work has found that excitatory stimulation over the right DLPFC decreases cravings for unhealthy foods, and in some cases this stimulation pattern also decreased actual consumption.

Why does tDCS seem more likely to reduce craving but not consumption as the studies above suggest? It may be that cravings or subjective responses are easier to modulate with tDCS (and other interventions) than consumption or behavior. The fact that more of the studies reviewed above find craving effects than consumption effects is consistent with this view.

In the case of studies where cravings are reduced but not reduced consumption, in these studies it may be the case that craving was not reduced *enough* to reduce eating. Additional consumption without craving (or low levels of craving) may reflect a form of dysregulation suggesting more automatic/habitual factors may influence consumption independent of craving (e.g., mindless eating). These reasons suggest that many contextual factors may make detecting links between impulse and consumption more difficult.

### Impulse Control

Impulse control involves the inhibition of emotive response tendencies. It plays a key role in diverse behavioral domains including emotion regulation, prosocial behavior, and risk taking.

#### Emotion Regulation

Emotions are not always functional. For example, emotions may work against one’s goals when they are expressed at the wrong time or at an inappropriate level of intensity (Taylor and Liberzon, 2007). Emotion regulation refers to the conscious and non-conscious processes individuals use to influence the intensity, variety, and duration of their emotions (Gross, 1998, 2001). Antecedent-focused emotion regulation strategies aim to modulate emotional responses before they solidify, whereas response-focused strategies aim to modulate emotional responses that have already been initiated (Gross, 1998, 2001). Neurobiological models of emotion regulation distinguish between bottom-up emotion regulation (e.g., expressive suppression) mediated by limbic system structures, such as amygdala, and top-down emotion regulation (e.g., cognitive reappraisal), which involves prefrontal regions (e.g., Ochsner and Gross, 2007).

Current neural models of emotion regulation (e.g., Phillips, 2003; Phillips et al., 2008) further distinguish between deliberate, conscious emotion regulation processes, which involve specifically the DLPFC, and more automatic, implicit regulation, which predominantly recruits the VLPFC (Chaiken and Trope, 1999; Strack and Deutsch, 2004; Mauss et al., 2007). Although emotion regulation typically recruits a wide range of cortical and subcortical structures, the DLPFC and the VLPFC constitute two central hubs in emotion regulation processes. These two regions contribute also to other forms of self-control, including motor control, risk-taking behavior regulation, task switching, response inhibition, and conflict monitoring (Braver et al., 2003; Aron et al., 2004; Berkman et al., 2009).

Evidence from fMRI research regarding the contributions of VLPFC and DLPFC to emotion regulation paved the way for the application of non-invasive brain stimulation to these regions to investigate stimulation effects on both explicit and implicit control strategies. For instance, one study tested the hypothesis that a top-down form of antecedent-focused emotion regulation – cognitive reappraisal – involves DLPFC. Indeed, anodal tDCS over the right DLPFC combined with a cognitive reappraisal task reduced negative emotions (but not positive emotions or craving) compared to anodal tDCS over the left hemisphere and sham stimulation (Pripfl and Lamm, 2015). This pattern of finding corroborates the widely established role of the right hemisphere in painful or aversive feelings (Canli et al., 1998; Kalisch et al., 2006; Baek et al., 2012; Herrmann et al., 2016, 2017; Salas et al., 2016; Mattavelli et al., 2017), but in this case stimulation to increase activity over right DLPFC reduced negative emotions. This perspective is further supported by the evidence that anodal tDCS over the right VLPFC, compared with sham stimulation, reduces the perceived intensity of negative (i.e., fear, anxiety, and sadness) but not positive or neutral emotions induced by film clips (Vergallito et al., 2018). In this study, participants were not explicitly instructed to apply any regulation strategy, suggesting a role for the VLPFC in incidental or implicit emotion regulation. In the next paragraphs we review tDCS effects on negative emotions, empathy, and social pain. Most of the reported studies have tested tDCS application on prefrontal cortex, mainly on DLPFC and VLPFC.

#### Anger and Aggression

Anger and aggression are associated with the activation of the behavioral approach system (Harmon-Jones and Sigelman, 2001; Harmon-Jones, 2003). The behavioral approach system is associated with greater left than right frontal cortical activity (Wacker et al., 2008; Zinner et al., 2008; Harmon-Jones et al., 2010). Using tDCS in laboratory aggression paradigms, several researchers have found additional support for a causal relationship between greater relative left frontal cortical activity and approach motivation.

Hortensius et al. (2012) asked participants to write a short essay on a controversial topic (e.g., abortion) before receiving insulting feedback on their essay from another ostensibly real participant. After writing the essay but before receiving the insulting feedback, participants received 15 min of tDCS. By random assignment some participants received stimulation to increase in relative left frontal cortical excitability (anodal over F3/cathode over the F4), increase in relative right frontal cortical excitability (cathode over F3/anode over F4), or sham stimulation. After tDCS, participants played a competitive reaction time game against the purported insulter. The game was based on the Taylor aggression paradigm (Taylor, 1967). Aggression was operationalized as the duration and intensity of a noxious noise blast given to the other participant. Participants also reported how much anger they felt both pre- and post-insult. Results indicated that after receiving tDCS to increase relative left frontal cortical activity, individuals behaved more aggressively toward the other participant, but only when they also reported high insult-related anger. In other words, stimulation to increase relative left frontal activity strengthened the link between anger and aggression.

Dambacher et al. (2015b) also combined tDCS over the DLFPC with the Taylor aggression paradigm. They found that stimulation to increase relative right frontal activity reduced aggression. Taken together with the results of Hortensius et al. (2012), these results suggest that manipulating frontal asymmetry with tDCS can modulate aggressive behaviors in a manner consistent with previous correlational work linking aggression to relative left frontal asymmetry: increasing relative left frontal activity increases aggression (especially among angry individuals; Hortensius et al., 2012) whereas increasing relative right frontal activity reduces aggression using the same Taylor aggression paradigm (Dambacher et al., 2015b). Unfortunately, Dambacher and colleagues did not include a condition to increase relative left frontal activity, so, they were unable to test whether increased left frontal activity increases aggressive behavior, as was the case for angry individuals in the study by Hortensius et al. (2012).

One difference between the results of Hortensius et al. (2012) and Dambacher et al. (2015b) is that the former found a main effect of tDCS on aggressive behavior whereas the latter did not. Rather, Hortensius et al. found a moderated pattern of results whereby anodal stimulation over the left DLPFC/cathodal stimulation over the right DLPFC increased aggression only for those high in insult-related anger. The studies differed insofar as Dambacher et al.’s study included two experimental conditions, whereas Hortensius study included three conditions. Moreover, another study (Dambacher et al., 2015a) did not find an effect of stimulation condition on aggressive behavior. Although this latter study did include all three stimulation conditions as in Hortensius et al. (2012), stimulation occurred over the F7/F8 prefrontal regions, whereas Hortensius et al. and Dambacher stimulated over the F3/F4 prefrontal regions. Thus, methodological differences preclude a direct comparison of these studies. Despite modest support for the effect of asymmetrical frontal cortical activity on aggressive behavior, further research is needed.

One common response to negative emotions, like anger, is rumination, which is an automatic cognitive process characterized by repetitive and distressful thoughts (Denson et al., 2006; Wade et al., 2008). Parallel literatures on depression (e.g., Heller et al., 1995; Nolen-Hoeksema, 2000) and anger (e.g., Bushman, 2002) have developed competing hypotheses about how rumination relates to lateralized patterns of prefrontal cortical activity. The depression literature links rumination to an increase in right frontal cortical activity, whereas the anger literature links rumination to an increase in left frontal cortical activity.

A study by Kelley et al. (2013) tested these two competing hypotheses by delivering anodal or cathodal tDCS over the right or left frontal cortex while participants received negative (insulting) ratings on their essay writing. Results supported the rumination-depression literature in showing that anodal stimulation over the right frontal cortex, compared with the other stimulation conditions, caused enhanced rumination. This study left open the question of what this increase in rumination means. One option flowing from the rumination-depression literature is that an increase in right lateralized frontal brain activity reflects an increase in avoidance motivation; the stronger the avoidance motivation, the greater the rumination. Another possibility, which may align with the rumination-aggression literature, is that the increase in right frontal activity reflects an increase in inhibition rather than avoidance motivation. Therefore, when considering the increasing of right frontal cortex activity, anger is still experienced, but inhibited; conversely left frontal cortex activation is linked to an approach-oriented motivation. Consistent with the latter interpretation, Kelley et al. observed an association between rumination and behavioral inhibition sensitivity (BIS; Carver and White, 1994), which is thought to reflect inhibition more so than avoidance (e.g., Neal and Gable, 2017).

A follow-up study further explored the consequences of excitatory tDCS over the right DLPFC for inhibitory processing. A meta-analysis of neuroimaging studies using a Go/No-Go task found a broad pattern of right frontal cortical activation during response inhibition (Swick et al., 2011). A separate EEG literature has often found that right lateralized frontal EEG alpha asymmetry is associated with exaggerated avoidance-motivated reactions to aversive events (Tomarken et al., 1990; Dawson et al., 1992; Wheeler et al., 1993; Kalin et al., 1998; Coan et al., 2001). These literatures suggest competing hypotheses regarding the psychological correlates of activity in the right frontal lobe.

Kelley and Schmeichel (2016) used tDCS to test the competing hypotheses. One hypothesis, flowing from the frontal EEG literature, is that excitatory stimulation over the right DLPFC should increase avoidance motivation. A second hypothesis, flowing from the neuroimaging findings, is that excitatory stimulation over the right DLPFC should increase response inhibition. The key to differentiating these two hypotheses is to test the effects of stimulation over right DLPFC on the inhibition of avoidance-oriented impulses. Kelley and Schmeichel paired tDCS with an approach-avoidance task whereby participants enacted motive incongruent motor responses to appetitive and aversive IAPS images. Specifically, participants in the positive emotion condition pushed away positive images whereas those in the negative emotion condition pulled negative images toward them. These responses require inhibitory control insofar as avoiding rewards and approaching threats requires one to override a predominant response tendency.

The results revealed that anodal stimulation over the right frontal cortex facilitates the inhibition of both approach-incongruent and avoidance-incongruent responding. These results are not readily explained by an increase in avoidance motivation. Indeed, an increase in avoidance motivation would have slowed the enactment of an avoidance-incongruent response. But Kelley and Schmeichel (2016) found that stimulation over the right DLPFC sped up both avoidance- and approach-incongruent responding. This study thus supported the hypothesis that increasing right frontal cortical activity increases response inhibition.

#### Sadness

Several studies have concentrated on testing tDCS efficacy in sadness regulation, which has important implications for considering tDCS as a treatment device for major depression. Indeed, it has been widely observed that major depression is associated with an asymmetry in prefrontal cortical activity, specifically hypoactivity in the left DLPFC and hyperactivity in the right DLPFC. For instance, one study found that five sessions of bilateral, twice-a-day tDCS over the DLFC (anode left/cathode right) decreased self-reported sadness in a group of patients with severe, drug-resistant major depression. These improvements lasted up to a month after the end of the treatment (Ferrucci et al., 2009). These findings are in line with a growing body of evidence that tDCS can be effective for treating the symptoms of major depression (for a review, see Nitsche et al., 2009). Indeed, a randomized, double-blinded, sham-controlled study found mood symptoms ameliorations after five session of active prefrontal tDCS in a group of newly diagnosed patients (Fregni et al., 2006b). Moreover, a study by Boggio et al. (2009) found that a single tDCS session over prefrontal cortex, but not occipital or sham tDCS, improved accuracy in identifying emotionally positive visual material in a sample of participants with major depression.

#### Fear

Another major, consequential negative emotion is fear. Fear plays a crucial role in the onset and maintenance of chronic pain (Riva et al., 2014b) and several mental disorders, including anxiety and post-traumatic stress disorder (Mungee et al., 2014). Regarding fear regulation, one study using an unpredictable threat paradigm found that anodal tDCS over right prefrontal cortex decreased sustained fear and skin conductance levels in the context of unpredictable threats (Herrmann et al., 2017). This finding lends additional support to the idea that right PFC activation increases response inhibition rather than avoidance motivation. Another study, this one using a bipolar montage over the DLPFC with the anode over the left DLPFC and cathode over the right DLPFC, found improved reaction times on simple arithmetic decisions and decreased cortisol concentrations among participants high in math anxiety (Sarkar et al., 2014).

On the other hand, a recent tDCS study on the consolidation of fear memories observed that greater right frontal cortical activity may enhance fear-related responding. Mungee et al. (2014) paired a fear-conditioning paradigm with either cathodal stimulation (i.e., stimulation to decrease activity) over the right dorsolateral prefrontal cortex, anodal stimulation (i.e., stimulation to increase activity) over the right dorsolateral prefrontal cortex, or sham stimulation. Fear was measured via skin conductance responses to the conditioned stimulus. Results revealed that anodal stimulation over the right dorsolateral prefrontal cortex increased memory for the conditioned feared stimulus as measured via skin conductance responses. These results suggested that increasing activation of the right dorsolateral prefrontal cortex increases fear memory consolidation (see also Mungee et al., 2016). However, this study did not simultaneously pair anodal stimulation to the right dorsolateral prefrontal cortex with cathodal stimulation to the left dorsolateral prefrontal cortex to create an asymmetric pattern of activity, as was done in the studies described in the previous paragraphs in this section. Given that the effects of anodal stimulation over the right dorsolateral prefrontal cortex in conjunction with cathodal stimulation over the left dorsolateral prefrontal cortex have yet to be tested in the study of fear memory consolidation, the causal relationship between greater relative right frontal cortical asymmetry and avoidance motivation remains unclear. Collectively, the evidence on prefrontal tDCS effects on the regulation of fear-related responses remains mixed. Future studies should clarify the extent to which excitatory stimulation over the right DLPFC helps or hinders fear regulation.

#### Social Pain

Social pain refers to the hurt feelings caused by rejection or ostracism from the group. These aversive social experiences generally cause negative emotions and result in a loss of sense of belonging, control, and self-esteem (Williams, 2009). Some studies have found a role for the dorsal anterior cingulated cortex (dACC) and the right VLPFC in experienced and observed social exclusion conditions, suggesting the presence of a partially overlapping neural network involved both in physical and social pain (Eisenberger et al., 2003; Kross et al., 2007; Wager et al., 2008; Masten et al., 2009). The right VLPFC, in addition to being broadly involved in emotion regulation, has also been found to have a crucial role also in regulating the pain associated with social exclusion (Onoda et al., 2010).

These findings have lead researchers to test the effects of tDCS over brain regions involved in social pain regulation, mainly the right VLPFC. For example, participants in a study by Riva et al. (2012) received 15 min of anodal tDCS over the right VLPFC. At the end of the stimulation, participants were randomly assigned to be included or excluded in a virtual ball-tossing game called Cyberball (Williams et al., 2000). In the inclusion condition, subjects received a ball the same number of times as the other two players, whereas in the exclusion condition participants received the ball only on the first throws. Actually, participants did not play with real players; a computer program controlled the ball. Results revealed that anodal tDCS over right VLPFC, compared to sham stimulation, reduced levels of pain and hurt feelings among excluded subjects.

In a similar study, the same research group (Riva et al., 2014a) tasted the effects of tDCS on behavioral aggression following social inclusion or exclusion condition in the Cyberball paradigm. Participants received anodal or sham stimulation during the Cyberball game. At the end of the stimulation, subjects had to choose the amount of hot sauce for their ostensible partners to taste. This is the well-validated hot-sauce paradigm for studying aggression (e.g., Lieberman et al., 1999). Findings indicated that increasing right VLPFC cortical activity with tDCS reduced behavioral aggression among excluded participants, who were no more aggressive than included ones. These findings were replicated in a subsequent study (Riva et al., 2017), which found that brain polarization through anodal tDCS over the right VLPFC reduced unprovoked aggression as measured by the Taylor aggression paradigm (Taylor, 1967).

Similar results were obtained by Kelley et al. (2015a), who also paired a Cyberball game with prefrontal tDCS. In this modified version of the game, participants first chose a partner from a group of images of eight opposite-sex individuals. A third Cyberball player was assigned by the experimenter and was always the same sex as the participant. Harmon-Jones et al. (2009) had found that this Cyberball game evokes jealousy and that self-reported jealousy after being excluded by a desired partner correlates with relative left frontal cortical activity. Kelley et al. had participants choose a partner in the modified Cyberball paradigm and play a practice version of the game before receiving 15 min of tDCS in one of three conditions: excitatory left DLPFC stimulation (anode over F3/cathode over the F4), excitatory right DLPFC stimulation (cathode over F3/anode over F4), or sham stimulation. Stimulation to increase relative left frontal cortical activity increased self-reported jealousy. Because the direct manipulation of cortical excitability with tDCS produced the same outcome as the correlational finding reported Harmon-Jones et al. (2009), this study suggests that tDCS over the dorsolateral prefrontal cortex does indeed modulate emotive responses associated with social exclusion and asymmetric frontal cortical activity.

Collectively, these studies highlight the effects of tDCS over the prefrontal cortex on regulating responses to social exclusion. This work may have clinical implications for disorders characterized by maladaptive responses to social exclusion (e.g., borderline personality disorder). Future research should test the extent to which the increased negative affect and autonomic arousal (Kopala-Sibley et al., 2012) and inappropriate coping strategies and impulsive behaviors (Dixon-Gordon et al., 2011; Coifman et al., 2012) associated with these disorders can be modified by tDCS.

#### Empathy

Recently, some research has observed that personal physical suffering and empathy for the pain of others share the same neural network, involving prefrontal cortex as well as somatosensory cortex, anterior cingulate cortex, amygdala, and anterior insula (Eisenberger, 2012). To investigate the role of prefrontal cortex in empathic pain regulation, Boggio et al. (2009) conducted a study in which participants judged the unpleasantness of pictures showing human beings under painful conditions. Anodal tDCS was applied over the left DLPFC, the primary motor cortex, and the primary visual cortex (control site) in three experimental sessions. Findings revealed decreased unpleasantness and discomfort during anodal stimulation over left DLPFC compared to sham stimulation. Moreover, compared to a previous study in which tDCS was delivered together with an electrical peripheral stimulation (Boggio et al., 2008), no significant effects were found in the primary motor cortex stimulation condition, suggesting that DLPFC specifically contributes to the emotional processing of empathic pain.

These findings were expanded by a similar study comparing emotional reactions to negative, positive, and neutral human pictures (Peña-Gómez et al., 2011). This study found that anodal tDCS over the left DLPFC reduces only painful and not positive or neutral affects. Based on previously mentioned neural models (Phillips, 2003; Phillips et al., 2008; Kohn et al., 2014) and previous neuroimaging evidences regarding DLPFC involvement in more cognitive forms of emotion regulation (Blair et al., 2007; Boggio et al., 2007a; Ochsner et al., 2009; Peña-Gómez et al., 2012), the authors suggested that increasing activity in the DLPFC may enhance cognitive control of emotional reactions. Moreover, the tDCS effect was more noticeable for participants with higher subclinical scores on the introversion personality dimension, perhaps due to their enhanced ability to control emotion expression, which correlates with the increased cortical activity, compared with extraverts, especially in the frontal lobes (Suslow et al., 2010).

A similar finding was reported also by Feeser et al. (2014), who applied anodal tDCS over the right DLPFC while participants applied cognitive reappraisal strategies to down- or up-regulate the emotions elicited by negative or neutral pictures. Skin conductance responses were also assessed. Anodal tDCS, compared to sham stimulation, increased emotional and autonomic reactions when the reappraisal instruction was to upregulate emotions and decreased emotional and autonomic responses when participants attempted down-regulation. Taken together, the studies reviewed here implicate DLPFC and VLPFC in emotion regulation when observing or experiencing painful situations, thereby highlighting the feasibility of tDCS application for the study of pain and empathy for pain.

#### Risk Taking

In addition to emotion and mood manipulations, another way researchers have explored the consequences of prefrontal brain stimulation on emotion regulation is by studying risk taking. Risk taking involves the possibility of punishment or potential harm in the pursuit of rewards or goal-relevant actions. From an emotion-regulation perspective, risk-taking involves managing the emotions associated with the anticipation of winning and losing. In a seminal tDCS study, Fecteau et al. (2007a) used tDCS over the DLPFC during a risk task (Rogers et al., 1999). Across 100 trials of a gambling task, participants viewed 6 horizontal boxes. Some boxes were blue, and some were pink, and the ratio of blue to pink boxes varied from trial to trial. The ratio could be 5:1, 4:2, or 3:3. Of the two options, the high likelihood option was always associated with a small reward whereas the low likelihood option was always associated with a large reward. Participants were to indicate which color box contained a token. Each trial selecting the winning color box earned a reward but selecting the incorrect color box incurred a penalty. Larger rewards were always paired with riskier decisions such that correctly choosing a pink box with a low win probability (1/6) would result in a large reward whereas making that same choice and losing was associated with same magnitude of a loss. Thus, participant’s tendency to choose high-risk/unlikely rewards over low risk/likely rewards was the measure of risk-taking. Results indicated that excitatory right DLPFC stimulation increased the number of participants earned by decreasing risk taking on the task. These results suggest that excitatory stimulation over the right DLPFC tilted participants toward safer, less risky choices, as though they were less tempted by the larger, riskier rewards.

These findings have been partially replicated by Cheng and Lee (2016), who found that the excitatory stimulation over the right DLPFC influences performance on the Risky Gains Task (RGT; Paulus et al., 2003), but not on the Balloon Analogue Risk Task (BART; Lejuez et al., 2002). Regarding the RGT task, participant’s goal was to win as many points as possible. To accomplish this goal, they made quick (1 s) decisions between taking a reward (i.e., points) now or waiting for a larger point value later. However, this later-but-larger reward also came with the risk of being punished (i.e., losing points equivalent to the later-but-larger reward). Risk-taking was quantified as the rate at which participants selected trials for which punishment was possible whereas safe decision-making was the rate at which participants chose point values with no possibility of punishment. The effect of excitatory right DLPFC stimulation on risky (but not safe) trials was moderated by individual differences in impulsivity, such that greater risk-taking was associated with greater (versus lower) impulsivity. More recently these results have recently been conceptually replicated in a clinically impulsive sample. Gilmore et al. (2017) paired excitatory stimulation over the right DLPFC (twice a day for 5 days) with a balloon analog risk taking (BART) task. They found that excitatory stimulation over the right DLPC reduced risk-taking by 46%. This diminished risk-taking persisted at a two-month follow-up.

Another study probed the underlying neurocircuitry that may be driving the changes in risk taking. Weber et al. (2014) used excitatory stimulation over the right DLPFC, prior to functional MRI during which participants completed the BART. They found that excitatory stimulation over the right DLPFC increased activity in the right DLPFC and the ACC as well. Additionally, this pattern of stimulation also influenced how these two regions connected with the rest of the brain. Specifically, greater right DLPFC-whole brain connectivity was associated with diminished risk-taking on the BART. These results suggest that the diminished risk-taking linked to excitatory right DLPF stimulation influences the neurocircuitry implicated in successful self-regulation.

In addition to laboratory risk-taking paradigms, an innovative study paired prefrontal tDCS with a more ecologically valid form of risk taking: driving behavior. In a driving simulator study, Beeli et al. (2008) observed that excitatory stimulation of the DLPFC (either left or right) led participants to keep a safer distance behind a lead driver and reduced the number of speeding errors. This pattern of stimulation did not influence average speed or revolutions per minute. These results are broadly consistent with reduced risk taking, but they differ from the risk-taking findings reviewed above in a crucial way: the electrode montage used. Whereas the risk-taking studies described above paired excitatory stimulation over the right DLPFC with inhibitory stimulation over the left DLPFC, the study be Beeli and colleagues placed the cathode over the ipsilateral mastoid. Because of this difference in electrode montages it is unclear to what extent these results are comparable to the risk-taking studies above. Future studies should explore the extent to which anodal right DLPFC/cathodal left DLPFC stimulation influences risk-taking in this domain.

In summary, numerous studies have observed enhanced self-regulation after anodal tDCS over right DLPFC (cathodal over left). Improved self-regulation manifested in a variety of ways including greater pain tolerance, healthier food choices, less impulsive decision-making, improved emotion regulation, reduced anger and aggression, less sadness and fear, more empathy, and less risk taking. Collectively, these results anodal tDCS over right DLPFC may be one tool that shows promise in helping individuals live happier, healthier lives.

### Limitations and Future Directions

We have reviewed evidence suggesting that non-invasive brain stimulation over the prefrontal cortex may improve human self-regulation. First, we detailed a methodological review of tDCS highlighting that tDCS is a safe, inexpensive, and easy to use technique that can be used to study higher-order cognition, emotion, and clinical phenomena. We also reviewed stimulation parameters that may help or hinder tDCS efficacy including current density, stimulation duration, and the orientation and focality of the electrical field. Second, we reviewed findings from experiments observing that non-invasive brain stimulation over the PFC can enhance three paradigmatic forms of self-regulation: persistence, delay behavior, and impulse control. Although the current review focused predominantly on tDCS, other techniques such as low energy neurofeedback (e.g., Ochs, 2006) and transcranial ultrasound (e.g., Tufail et al., 2011) also represent low-cost, non-invasive techniques for investigating the causal role of neural activation in various forms of self-regulation.

Although much of this review highlighted the ability of brain stimulation to promote self-regulation, under the right circumstances brain stimulation may also undermine self-regulation. For example, we highlighted studies wherein excitatory stimulation over the right DLPFC enhances inhibition (e.g., Kelley and Schmeichel, 2016) whereas excitatory stimulation over the left DLPFC increases approach motivated negative emotions like anger (e.g., Hortensius et al., 2012) and jealousy (e.g., Kelley et al., 2015a, b). These contrasting findings imply that excitatory tDCS is not an unmitigated good that improves self-regulation in all cases. In fact, these findings emphasize the need to identify precise stimulation parameters that help versus hinder self-regulation. Below, we discuss inconsistencies, unresolved questions, and new directions emanating from the above review.

#### tDCS Dosage

The efficacy of eliciting changes in brain activity using tDCS depends on several physical parameters including current density, stimulation duration, and the orientation and focality of the electrical field. These parameters constitute the tDCS dosage. As we noted above, different electrode positions modulate different neuronal groups and elicit different evoked potentials in the case of primary motor cortex and primary visual cortex stimulation, respectively (Priori et al., 1998; Antal et al., 2004; Accornero et al., 2007), and these different electrode positions may also determine effects on the targeted brain region due to electrical field interactions associated with neuronal geometry (Nitsche and Paulus, 2000; Nitsche et al., 2008).

Whereas the coupling of anodal-excitatory and cathodal-inhibitory effects is well established in the sensorimotor domain, the evidence pertaining to higher cognitive functions is more controversial (Jacobson et al., 2012). Indeed, when dealing with more complex functions represented by large and interconnected neural networks comprising both excitatory and inhibitory connections, it is more difficult to obtain predictable outcomes – especially when different electrode montages have been used across studies. Hence it is not always the case that anodal tDCS leads to an enhancement (e.g., better performance in a task) and cathodal tDCS leads to a diminution of the assessed cognitive function (Fertonani and Miniussi, 2017).

To illustrate this point, consider the studies above on physical pain tolerance. These studies found increased pain tolerance as the result of excitatory stimulation of the right DLPFC (Mylius et al., 2012), excitatory stimulation over the left DLPFC (Boggio et al., 2008; Mariano et al., 2016), and inhibitory stimulation over the left DLPFC (Powers et al., 2018). Of the four studies we reviewed, three difference electrode montages were used. Notably, the study by Powers and colleagues, unlike the other pain studies, paired tDCS with clinical interventions. These methodological differences likely contributed to the differences in results across studies and thus makes interpreting the effects of tDCS difficult. Future studies should more precisely optimize the stimulation protocols that accentuate versus undermine self-regulation for easier comparison across studies.

More broadly, however, the findings from the pain studies are congruent with the findings from studies of other self-regulatory domains in suggesting that stimulation over right DLPFC facilitates self-regulation. Whether such stimulation is most profitably paired with inhibitory (cathodal) stimulation over left DLPFC remains to be seen. And the effects of excitatory (anodal) stimulation over left DLPFC on self-regulation are even more uncertain, with some studies finding better and some finding worse self-regulation after excitatory stimulation over left DLPFC.

#### Individual Differences

Another inconsistency in past research concerns individual differences. Despite seminal work suggesting that participants should be matched on individual differences like sex or age that can influence tDCS efficacy (Pitcher et al., 2003; Kuo et al., 2006; Quartarone et al., 2007; Chaieb et al., 2008; Nitsche et al., 2008), many of the studies reviewed above did not use this strategy, and we recommend future studies implement matching procedures to reduce error variance and increase power to detect tDCS effects.

In addition to demographic factors, which may subtly influence tDCS efficacy, many of the studies we reviewed also did not consider the extent to which participants are responsive to tDCS. Previous research has found that between 50 and 64 percent of participants are responsive to tDCS protocols, which leaves a substantial proportion of individuals who are not particularly responsive to tDCS (Wiethoff et al., 2014; López-Alonso et al., 2015). Studies that include non-responders and studies that do not systematically test for differences between responders and non-responders many obscure effects of tDCS on regulatory behavior. Further, only few studies have considered individual differences traits, though these are also likely to moderate tDCS effects. For example, the study by Kekic et al. (2014) found that tDCS-driven reductions in food craving were more pronounced among participants with low (versus high) delay discounting tendencies. Evidence of this sort highlights the need to develop more robust stimulation protocols in well-powered studies that can systematically test for the moderating role of individual differences.

#### Impulse-Behavior Relationship

Another inconsistency in the research reviewed above concerns whether changes in emotive responding extend to changes in behavior. We summarized evidence that tDCS may alter food craving without altering food consumption (although sometime consumption changes, too; e.g., Fregni et al., 2008). Similarly, we summarized evidence that changes in emotional responses (e.g., anger) may occur in both the absence of and presence of corresponding changes in behavior.

The path from impulse to behavior entails multiple determinants, and thus impulses and behaviors may diverge for several reasons. Practical considerations including variability in stimulation dosage may explain why tDCS sometimes does and sometimes does not influence behavior. For example, one important aspect of the tDCS dosage is electrode size. The study by Kekic et al. (2014) who did not find effects on food consumption used 25 cm^2^ electrodes whereas Fregni et al. (2008) used 35 cm^2^ electrodes. Smaller electrodes are more spatially precise compared to larger electrodes. The larger electrodes may affect distal brain regions that are less affected in studies using smaller electrodes possibly accounting for why the study by Fregni et al. founds effects on consumption, but Kekic did not. Beyond these practical considerations idiosyncratic food preferences may have played a role in the inconsistent results. The studies assessing compulsion offered actual food to participants, but those foods were not specifically tailored to the participant. Instead participants were offered foods that are generally (but not universally) well liked, such as chips, cookies, and chocolate. This may be problematic insofar as some participants may not prefer any of the food options offered by the experimenter. Future studies using more ecologically valid measures of food consumption and others types of regulatory behavior (e.g., naturalistic observation) may prove useful in clarifying the extent to which tDCS-induced changes in emotions/impulses influence subsequent behavior. However, we must also consider the possibility that cravings/subjective states are more readily influenced by tDCS, whereas behaviors may be harder to change as they have a multiple of determinates above and beyond the preceding subjective state.

#### How Does tDCS Effect Underlying Brain Activity?

The effects of tDCS on underlying brain activity are not well understood and subject to ongoing debate. It remains to be seen how tDCS affects brain activity and how these changes relate to changes in self-regulation. One way to advance research on this topic is to pair concurrent measures of brain function with electrical stimulation. Candidate neural processes mediating links between tDCS and self-regulation include changes in prefrontal EEG alpha and functional connectivity between the prefrontal cortex and subcortical regions.

#### Prefrontal Alpha

One potential mediator driving many of the effects reported in the current review is EEG alpha activity. Specifically, lateralized patterns of alpha activity may reflect a person’s motivational orientation with left lateralized EEG alpha activity reflecting approach-motivation (Tomarken et al., 1992; Harmon-Jones and Allen, 1997, 1998; Sutton and Davidson, 1997; Harmon-Jones and Sigelman, 2001; Harmon-Jones et al., 2002, 2006; Coan and Allen, 2003; Harmon-Jones, 2007) and right lateralized pattern of EEG alpha activity has been linked to withdrawal or avoidance motivation (Davidson et al., 1990; Tomarken et al., 1990; Dawson et al., 1992; Kalin et al., 1998; Coan et al., 2001). Self-regulatory failure tends to occur when individuals have strong impulses such as strong impulses to engage in approach or avoidance motivated behavior. Likewise successful self-regulation tends to occur when impulses are weaker. To the extent that asymmetric patterns of alpha activity in the frontal cortex reflect the strength of appetitive and aversive impulses they may be a good candidate linking tDCS to changes in self-regulation.

Indirect support for the role of EEG alpha activity in tDCS effects comes from parallel findings using EEG and tDCS, respectively, to study the emotion of jealousy. Harmon-Jones et al. (2009) found that feelings of jealousy during a Cyberball game correlate with greater left frontal alpha activity, and Kelley et al. (2015a,b) found the same pattern by manipulating (rather than measuring) brain activity with tDCS. More specifically, Kelley et al. observed that excitatory stimulation of the right DLPFC paired with inhibitory stimulation of the left DLPFC increases feelings of jealousy during a Cyberball game. By finding the congruent effects with both measured and manipulated brain activity, the pair of results together suggest that EEG alpha activity may mediate the link between prefrontal tDCS and behavioral self-regulation.

In an innovative new study, Vöröslakos et al. (2018) developed an intersectional short-pulse (ISP) stimulation paradigm in cadaver and rodent studies. ISP delivers shorts bursts (less than 10 μs) of high intensity stimulation from multiple electrode pairs centered on a stimulation site of interest. ISP thus offers superior spatial focality plus higher current densities (7–9 MA) more easily tolerated than traditional tDCS paradigms (>2 MA), with relatively low charge densities and scalp sensations. Pairing Vöröslakos and colleagues then paired ISP with concurrent EEG measurement and observed that tDCS affects the amplitude of simultaneously recorded EEG alpha waves. This work provides an important example of how technical studies in mouse and cadaver models can be used to improve existing tDCS protocols. Future research should consider implementing innovate stimulation paradigms like ISP to increase the efficacy of existing stimulation paradigms and to trace the neural mediators of tDCS effects.

#### Functional Connectivity

The self-regulation findings we reviewed may also be mediated by frontal cortical-subcortical interactions. Due to its high spatial resolution, functional MRI is perhaps best suited to probe such possibilities. Research along these lines has already begun. Weber et al. (2014) administered excitatory stimulation over the right DLPFC prior to functional MRI of a risk-taking task. They found that excitatory stimulation over the right DLPFC influences how the right DLPFC connects to the rest of the brain. Specifically, stronger right DLPFC-whole brain connectivity and diminished risk-taking emerged in participants who had received excitatory stimulation over the right DLPFC. Although this study did not measure brain function during tDCS, the finding that tDCS administered immediately prior to a risk-taking task influences connectivity patterns revealed by fMRI does highlight that stimulation may influence communication among neural networks. Future studies should continue to probe the extent to which prefrontal stimulation influences connectivity to reward-relevant (e.g., ventral striatum) and threat-relevant (e.g., amygdala) subcortical regions.

Potential neural aftereffects of tDCS, such as changes in prefrontal alpha asymmetry or functional connectivity, may mediate the relationship between tDCS and regulatory behavior. What remains to be established is the extent to which alpha asymmetry or functional connectivity carry the causal effects of tDCS on self-regulation, or whether they merely correlate with observed changes in regulatory behavior.

#### How Do Impulse and Control Strength Contribute to Regulatory Behavior?

Imagine a person who, when offered a chocolate treat, decides not to eat it. Does foregoing chocolate reflect the application of control to resist chocolate consumption, a low desire for chocolate, or some combination of both things? Social psychologists have traditionally viewed self-regulatory success as the consequence of applying self-regulatory strength (see Baumeister et al., 1994, 1998, 2007). In this view, a person forgoes chocolate by resisting the temptation to eat it. But impulse strength also contributes to self-regulatory outcomes. Presumably, weaker impulses are easier to control or hardly require control at all (e.g., Inzlicht and Schmeichel, 2012; Kelley et al., 2018). Successful self-regulation has been associated both with increased activity in areas associated with top-down control, including the prefrontal cortex, and reduced activity in subcortical regions involved in reward and threat processing. These patterns also implicate and potentially confound strong control and weak impulses. Thus, an unresolved issue concerns the relative contribution of control strength and impulse strength to self-regulatory outcomes. By paring tDCS with measures of concurrent brain function, it may be possible to compare the contributions of neural activity caused by (1) stimulation to areas associated with impulse strength, and (2) stimulation to increase activity in control regions.

#### What Are the Long-Term Consequent of Brain Stimulation on Regulatory Behavior?

In addition to limited understanding of the effects of tDCS on underlying neural processes, we also have a limited understanding of the long-term effects of tDCS. Most of the studies we reviewed involved just a single session of tDCS. Although some studies have involved the administration of tDCS once a day for multiple days (Fregni et al., 2006a; Boggio et al., 2007b), those studies have primarily been conducted with patients recovering from spiral cord injury or stroke. Pairing repeated stimulation with measures of self-regulation in non-patient samples may yield stronger, more consistent effects.

Self-regulation research touches on alcohol and drug addiction, personal debt, obesity, and a variety of other consequential outcomes. It is important to acknowledge that these outcomes are not the result of one decision or behavior but instead results from multiple patterns of behavior unfolding over long periods of time. Few studies have examined long-term downstream effects of stimulation. One notable exception was a study by Gilmore et al. (2017), who found that excitatory stimulation over the right DLPFC (twice a day for 5 days) reduced risk-taking at the end of the stimulation period and at a two-month follow-up. We encourage future studies to follow the example of Gilmore and colleagues and use both repeated stimulation paradigms and longitudinal designs to examine changes in behavior. Extend this work to include also concurrent changes in brain function would help to more precisely map the long-term consequences of tDCS on regulatory behavior.

## Conclusion

Self-regulation is crucial for goal-directed behavior and contributes to many consequential outcomes in life including physical and mental health, decision making, and interpersonal functioning. Likewise, failure at self-regulation is thought to contribute to alcohol and drug addiction, personal debt, obesity, and other outcomes that carry both personal and societal costs. Based on the evidence reviewed here, we conclude that brain stimulation techniques hold promise for improving self-regulation across three broad domains of response: persistence, delay behavior, and impulse control. To maximize the utility of tDCS in future research, we encourage researchers be especially mindful of issues such as dosage and individual differences – issues that have been commonly been neglected in past research and have likely contributed to inconsistencies in past findings. Future research that takes these concerns into account and incorporates concurrent measures of brain activity will bring the promise of tDCS into sharper focus and help to reveal underlying neural mechanisms.

## Author Contributions

All authors contributed to the conception, writing, and revising of this manuscript.

## Conflict of Interest Statement

The authors declare that the research was conducted in the absence of any commercial or financial relationships that could be construed as a potential conflict of interest.
